# (U,Th)-He dating of Pleistocene carbonates: Analytical methods and age calculations

**DOI:** 10.1016/j.mex.2021.101608

**Published:** 2021-12-18

**Authors:** Jan D. Kramers, Tebogo V. Makhubela

**Affiliations:** PPM Research Centre, Geology Department, University of Johannesburg, South Africa

**Keywords:** Speleothem dating, (U,Th)-He dating, Helium dating, U/Th disequilibrium, Hominin evolution timeline

## Abstract

The uranium-thorium-helium ((U,Th)-He) dating method applied to calcium carbonate speleothems holds much promise for constraining the timeline of hominin evolution, as well as for palaeoclimatology research beyond the range of U/Th disequilibrium dating. Technical problems are posed by often low U concentrations and the requirement that samples need to be individually removed from the ultrahigh vacuum (UHV) system after helium is extracted from them, to be then analyzed for U, Th content and U series disequilibrium. We describe a low-cost furnace with this capability, constructed from standard UHV components, as well as the methods for subsequent U/Th disequilibrium analysis using multicollector ICP mass spectrometry. We present analytical and numerical solutions to determine (U,Th)-He ages for four conditions that have been encountered: (1) for an age range in which the residual activity ratio (^234^*U*/^238^*U*) can still be resolved but not that of (^230^*Th*/^234^*U*) (up to about 3000 ka), (2) for an age range where neither can be resolved (unlimited), (3) for ages up to 1000 ka where both activity ratios may be resolvable, and (4) for cases where (^234^*U*/^238^*U*) and (^230^*Th*/^234^*U*) indicate ages < 200 ka but (U,Th)-He systematics point to much older ages.

• Helium extraction is carried out using an in-house built vacuum furnace that allows for large sample sizes (20 to 100 mg) of powdered carbonate material wrapped in Cu foil.
• U and Th are separated together using Eichrom UTEVA^Ⓡ^ resin and their isotope abundances are measured together in a single 2-cycle dynamic run using the multicollector inductively coupled plasma mass spectrometer (MC-ICP-MS).
• (U,Th)-He ages are calculated using four methods that take into account the U/Th disequilibria.

## Graphical abstract

**Figure:**
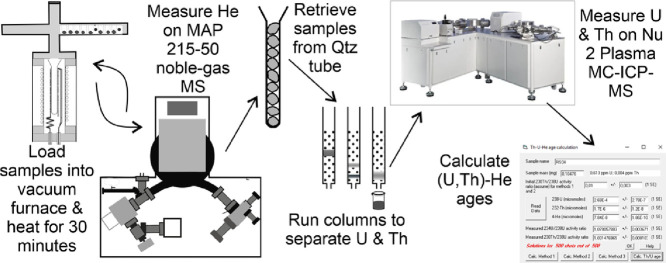

Specifications Table

**Table:** 

Subject Area:	*Earth and Planetary Sciences*
More specific subject area:	*Geochronology of Plio-Pleistocene speleothems.*
Method name:	*- Helium extraction from carbonate samples with post-extraction sample retrieval.*
	*- U,Th isotope measurements in a 2-cycle dynamic measurement on a multicollector ICP-MS.*
	*- Numerical methods for (U,Th)-He age calculation with U/Th disequilibrium.*
Name and reference of original method:	*Various helium dating methods for carbonates including Fanale & Schaeffer (1965, Science, 149, 312–316), Bender (1973, Geochimica et Cosmochimica Acta, 37, 1229–1247), and Copeland et al. (2007, Geochimica et Cosmochimica Acta, 71, 4488–4511).*
Resource availability:	*Requires both noble gas mass spectrometry and either TIMS or Multicollector ICP mass spectrometry (not necessarily in the same place)*

## Introduction

This is a companion paper to Makhubela and Kramers [1] on combined (U,Th)-He and U/Th dating of Plio-Pleistocene calcite speleothems. Common applications of the (U,Th)-He dating method include thermochronological research, essentially on apatite and zircon [2], [3], [4], [5], [6] as well as research on ore deposits and landscape evolution where hematite and goethite are normally analyzed [7,8]. All (U,Th)-He applications require extraction and mass spectrometric analysis of He in ultrahigh vacuum (UHV), followed by analysis of U and Th (and in rare cases Sm) in the same sample aliquot. Samples for thermochronology are normally U- and/or Th rich, and in both the economic geology and the thermochronology applications, the age range considered is far beyond that in which U/Th disequilibria can still be measured. Therefore, only U and Th concentrations are normally analyzed post He extraction. Thus, small aliquots (ca. 1 mg or less) suffice for an analysis, enabling heating by laser, which renders sample retrieval after He extraction unproblematic.

In contrast to the matter analyzed in these common applications, calcite speleothems have low U and Th concentrations (generally below 1 ppm), and young ages, both of which lead to low He content. Further, in young carbonates residual disequilibria in (^234^*U*/^238^*U*) and even (^230^*Th*/^234^*U*) activity ratios (hereinafter shown in brackets) can still be detectable, and must be considered in age calculations. These factors, combined, require relatively large sample sizes (20 to 100 mg), beyond the capability of most external heating by lasers or halogen lamps. Thus, a vacuum furnace is needed from which samples can be individually removed after He extraction, without having to break the vacuum after each sample.

A further issue is the calculation of (U,Th)-He ages where ^238^U, ^234^U and ^230^Th are not in secular equilibrium. This is more complicated than calculating U-Pb ages with the same condition, because unlike ^206^Pb, He is not only yielded as an end product of ^238^U decay, but as α particles between ^238^U, ^234^U and ^230^Th (1 each) as well as between ^230^Th and ^206^Pb (6 α particles). Including the disequilibrium in an age determination is in this case not a simple correction but must be integrated in the calculation itself.

## He extraction procedure: Furnace, sample magazine and cold trap design

**Fig. 1:**
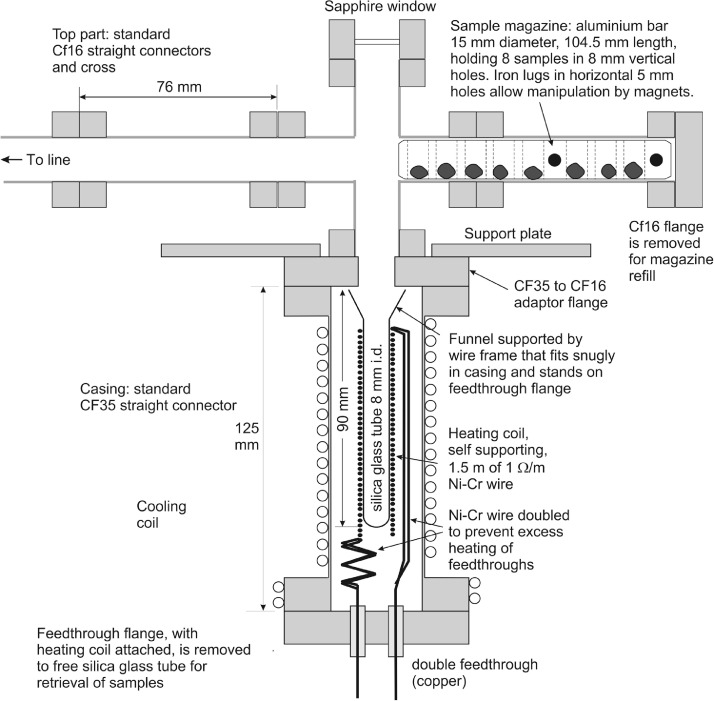
The furnace used for helium extraction, constructed from standard CF UHV components. Samples in Cu capsules must have diameters 6 mm or larger, to avoid samples falling past each other in the silica glass tube.

The furnace constructed for this purpose and its sample magazine holding 8 samples, are shown in Fig. 1. A silica glass sample tube (10 mm outer and 8 mm inner diameter) with a funnel-shaped top (top diameter 20 mm) is surrounded by a heating coil made from Ni-Cr alloy wire of resistance 1 Ω/m, of 1.5 m total length. This coil has sufficient mechanical rigidity not to require support other than the base, provided by the high current feedthrough. The sample tube is suspended from its funnel top by a wire frame that fits snugly in the casing but can be easily extracted after the heating assembly (coil and feedthrough flange) are removed.

The sample magazine of the furnace cannot be of the usual type (glass tree with magnetic control of sample introduction through iron lugs) because of He leakage through the glass. Carousels can be used but these are costly. A linear sliding sample magazine, integrated in the furnace design, was constructed (Fig. 1), which consists of a 15 mm diameter aluminum bar. Samples, wrapped in copper foil, are placed in vertical 8 mm diameter holes through the bar. They are individually introduced into the furnace by moving the bar using magnets, while observing its position through the sapphire window.

Samples are heated to ca. 950˚C, well below the melting point of Cu (1085˚C) and well above the temperature of calcination (CaCO_3_ → CaO + CO_2_; ca 800˚C, [9]). We found that even with powdered samples, ca. 30 minutes are required to fully calcine the samples and extract all the He at this temperature. This high temperature and long duration required is surprising in view of published data on the diffusivity of He in carbonates [[10], [11], [12]]. To attain the same temperature independent of the number of samples in the furnace, the power supply is controlled via the glow emitted, using a phototransistor sensor in a cap placed over the sapphire window during heating. Before use, silica tubes are degassed into a turbo pump, and hot blanks are run before sample batches and interspersed with sample runs.

As very large amounts of CO_2_ are produced, no getters are open to the line during heating as these would be overwhelmed. Instead, a liquid nitrogen cold trap is used. This has a bottom diameter of 40 mm and contains 2 g of activated charcoal. Due to the large diameter, CO_2_ snow deposited on the cold trap wall does not block the way to the activated charcoal layer at the bottom, on which N_2_, O_2_ and Ar are largely adsorbed.

**Fig. 2:**
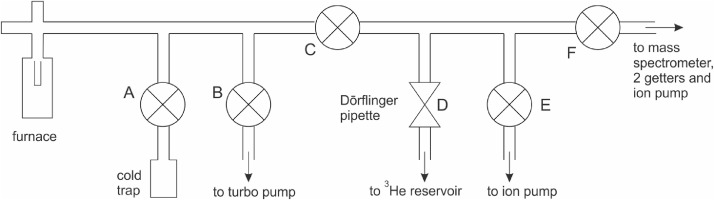
Schematic drawing of the extraction line used. Sequence of heating and valve operations given in the text.

A schema of the extraction line is shown in Fig. 2. This is part of a line also used for ^40^Ar/^39^Ar analyses and only the parts relevant to He analysis are shown. The line is made up of standard CF16 tubing (internal diameter 16 mm) and the valves are CF16 all-metal Varian-type angle valves. The mass spectrometer is a MAP-215-50 single collector instrument with a Johnston electron multiplier used in analogue mode. Twin SAES AP10 getters (one at room temperature and one at 450°C) are placed in a stainless-steel tube adjacent to the ion source, and the mass spectrometer has its own dedicated ion pump. The Dörflinger pipette has a volume of 0.2 ml and is connected to a 2.6 L reservoir containing ^3^He spike. The sequence of an analysis below refers to the valve labels (A to F) in Fig. 2.

1. Starting condition: line under UHV, cold trap is immersed in liquid nitrogen, valves B, E and F are closed, A and C open.
2. One pipette (D) volume of ^3^He spike is introduced into the line (this is done first, with the line empty, to avoid gradual contamination of the spike reservoir).
3. Sample is heated for ca. 30 minutes
4. Close valve A (to trap CO_2_ and adsorbed gases prior to volume increase in next step).
5. Open valve F and measure ^4^He/^3^He ratio.
6. Close valves C and F, open valve E and also pump out the mass spectrometer with its own ion pump.
7. Open valves A and B and immerse cold trap in boiling water to pump out CO_2_ and adsorbed gases with the turbo pump. Pressure usually goes up to the 10^−3^ mbar range for several minutes but reduces rapidly.
8. When pressure is down to the lower 10^−8^ mbar range, close valve B and open valves C and E, and immerse the cold trap back into liquid nitrogen to bring the system back to starting condition (1).

The ^3^He spike is calibrated by using a second 2.6 L reservoir, also connected to the line via a calibrated Dörflinger pipette (and normally used for air argon measurements). This reservoir is evacuated to <10^−7^ mbar, after which one pipette volume of pure natural He at recorded ambient temperature and atmospheric pressure is let into the reservoir. With the line again under UHV, the amount of He introduced into it from the reservoir via a single pipette volume can be precisely calculated. Spike calibration is done by measuring ^4^He/^3^He ratios after introducing pipette volumes of spike and natural He into the line. With this procedure there is no need to know the instrumental isotope fractionation, as this is canceled out between spike calibration and sample measurements. Following calibration of one ^3^He spike shot from the pipette (1.298 × 10^−6^ µmol), subsequent runs are counted, and the amount of spike is corrected for gradual depletion of the reservoir via an exponential equation. The ^4^He blank is between 7 × 10^−10^ and 1.5 × 10^−9^ µmol and the amount of ^4^He per sample generally ranges from 1 × 10^−7^ to 3 × 10^−5^ µmol. Blank corrections are in most cases not significant.

## Chemical separation and U, Th mass spectrometry

After extraction of He, the samples are removed from the furnace and transferred into 7 mL Savillex® vials. A ^229^Th + ^236^U double spike solution is added gravimetrically. Samples are dissolved in HCl or HNO_3_, along with the Cu foil capsule (to make sure that U or Th that may have been adsorbed onto the Cu foil is also dissolved). Where there is a residue of Fe-Mn oxide dust, this can be dissolved using concentrated HCl. Whatever acids are used for dissolution, it is important to take the samples fully to dryness and then fully convert them to the nitrate form by evaporating again with 5M HNO_3_ before column separation.

Ion exchange separation is done using Eichrom UTEVA® resin. Columns of inner diameter 3 mm and with 5 ml reservoir are filled to a height of ca. 6 cm above the frit, thus giving a resin volume of ca. 0.42 mL. Columns are pre-cleaned by passing through 3 × 3 mL of 1 N HCl and preconditioned by passing through 2 mL of 5 N HNO_3_. Samples are taken up in 2 mL of 5N HNO_3_ and loaded onto the columns, which are then washed through with 2 × 2 mL of 5 N HNO_3_. The washout is discarded. U and Th are eluted together with 2 × 2 mL of 1N HCl. Samples are taken to dryness at temperatures not exceeding 90°C. This is because Th, although a very refractory element, can adsorb on residual organic matter from the ion exchange resin and volatilize with this matter after the samples are dried if the temperature is too high. After drying, 60 µL of concentrated HNO_3_ and 20 µL of H_2_O_2_ are added (in that order) to oxidize any remnant of organic matter from the columns.

The way in which isotope U and Th abundances are measured in multicollector inductively coupled plasma mass spectrometers (MC-ICP-MS) depends on the type of instrument and its collector configuration. In our laboratory, U and Th isotopes are measured together in a single 2-cycle dynamic run on the Nu-II® MC-ICP-MS at the University of Johannesburg. The relevant part of the collector configuration and the cycle sequence are shown in Table 1.

**Table 1:** Collector use in U, Th isotope measurement on Nu-II MS, light side of the collector array (IC = ion counting electron multiplier, L = Faraday collector)

Collector	L3	L4	L5	IC0	IC1	IC2	L6	IC3	L7	IC4
**Cycle 1**		^238^U		^236^U	^235^U	^234^U				^230^Th
**Cycle 2**	^238^U		^236^U	^235^U	^234^U		^232^Th			^229^Th

Note: L3 to 6 are Faraday collectors, IC0 to 4 are ion counting electron multipliers.

The two measurements of ^235^U in IC1 and IC0 yield the gain ratio of these IC's. The two measurements of ^236^U in IC0 and L5 yield the gain of IC0 relative to the Faraday collectors. The measurement of ^234^U in cycle 1 is not used. As ^230^Th and ^229^Th are measured in the same IC, no gain factor plays a part in the measurement of their ratio. A gain for IC4 has to be measured only to obtain an accurate measurement for ^232^Th, which is usually not very critical. In order to compensate for plasma flicker, all individual IC readings are normalized to the ^238^U signal for the same cycle. U isotope ratios are primarily measured relative to ^235^U as this obviates the need for a precise gain measurement for IC0, and fractionation uncertainties are less critical than if ratios were measured against ^238^U. Mass fractionation, measured in separate runs using unspiked natural U, favours the heavier isotope and is best described by an exponential law. In our measurements, the exponential fractionation factor was c. 1.6, which for the U mass range corresponds to c. 0.4% per mass unit.

Originally, the MC-ICP-MS analyses were carried out from a 0.5 M HCl solution, using an ESR Apex-Q® desolvating nebulizer. HCl was used because this provided more efficient wash-out for Th than HNO_3_, using this nebulizer. However, in two early runs impossibly high apparent ^230^Th signals were measured (see [1]), which we ascribed to interference by PtCl (from atmospheric contamination by nanoparticles of Pt from automotive catalytic converters). Currently, the ultraclean Wits Isotope Geochemistry Laboratory (WIGL) laboratory at the University of the Witwatersrand is used for the chemical work, and measurements are done from 0.5 M HNO_3_ which avoids this problem. A simple cyclone spray chamber is now used wherein the very fast and complete washout makes up for the smaller signal.

## (U,Th)-He age calculations and disequilibrium corrections

The feasibility study of [1] comprised speleothems ranging in age from 100 to 3000 ka, and corrections for deviation of initial (^234^*U*/^238^*U*) and/or (^230^*Th*/^238^*U*) activity ratios from secular equilibrium may thus be significant. The classical analysis of the abundance of intermediate nuclides in a decay chain in the approach to secular equilibrium [13] has been extended to (U,Th)-He dating of young volcanic rocks where (^230^*Th*/^238^*U*)_initial_ may deviate from unity [14,15] and to corals, where normally (^234^*U*/^238^*U*)_initial_ >1 and (^230^*Th*/^238^*U*)_initial_ ≈ 0 [16]. The latter is generally also assumed in U/Th dating of speleothems, but several cases of (^230^*Th*/^238^*U*)_initial_ >> 0 were observed in our companion paper [1]. Different age ranges and other properties of samples were seen to require different approaches in age calculations. Four methods can be used, which are summarized in Table 2.

**Table 2:** Methods for (U,Th)-He calculations used.

Method #	Approx. age range (ka)	Initial (^230^Th/^238^U)	Initial (^234^U/^238^U)	Detrital correction
1	1000 - 3000	assumed	inferred	no
2	> 3000	assumed	assumed	no
3	< 1000	inferred	inferred	no
4	< 500	inferred	inferred	yes

Method 1 is applied to speleothems in the age range from c. 1000 ka to c. 3000 ka. The (^230^*Th*/^234^*U*) activity ratio is expected to be ≈ 1 (equilibrium) for any realistic initial values of this ratio. The measured (^234^*U*/^238^*U*) activity ratio can still be resolved from unity, which allows the initial value to be calculated (see Eq. 3 below). The initial (^230^*Th*/^234^*U*) is normally assumed to be close to zero.

Method 2 is used for samples older than ca. 3000 ka, where (^234^*U*/^238^*U*) is in secular equilibrium, or in cases where its measurement is imprecise. Initial values are assumed for both (^234^*U*/^238^*U*) and (^230^*Th*/^238^*U*). In the Cradle of Humankind caves, initial (^234^*U*/^238^*U*) ratios between 1 and 6 have been reported in U/Th and U-Pb studies, and thus random values in that range are input. Using a Monte Carlo approach to error propagation (see below), this means that all values in that range have equal probability.

Method 3 is applied to samples younger than ca. 1000 ka, where measured values of both (^234^*U*/^238^*U*) and (^230^*Th*/^238^*U*) may be used to constrain their initial values, and together with the He and U contents, this allows calculation of the age without any assumptions.

Method 4 addresses a condition in which disequilibrium in (^234^*U*/^238^*U*) and (^230^*Th*/^234^*U*) activity ratios point to young ages (e.g. < 200 ka), while the He, U and Th abundances indicate much older ages (e.g. > 500 ka). This is encountered particularly in calcretes but also in some speleothems. In this case the old (U,Th)-He apparent age points to a significant, much older component, which can therefore be expected to be at or near secular equilibrium. Then the U/Th age can be calculated after a conventional detritus correction, i.e. subtraction of a component with (^234^*U*/^238^*U*) = (^230^*Th*/^238^*U*) = 1, the amount of which is derived from the ^232^*Th*/^238^*U* ratio of the sample and an assumed ^232^*Th*/^238^*U* ratio for the old component.

All ages in this work and the companion paper [1] are in ka (thousand year) units. The values of the decay constant/ka are:

λ_238U_ = (1.55125 ± 0.00166) × 10^−7^ [17]

λ_235U_ = (9.8485 ± 0.0134) × 10^−7^ [17]

λ_232Th_ = (4.94752 ± 0.00495) × 10^−8^ [18]

λ_234U_ = (2.8222 ± 0.0030) × 10^−3^ [19]

λ_230Th_ = (9.1706 ± 0.01335) × 10^−3^ [19]

To calculate the accumulation of ^4^He in matter with initial (^234^*U*/^238^*U*) and (^230^*Th*/^238^*U*) disequilibrium, we have used the solution of Bender [16], with a modification to accommodate the possibility that (^230^*Th*/^238^*U*)*_init_* ≠ 0. In this solution, isotope abundances are expressed in terms of activity ratios, which is advantageous when considering ^4^He abundances together with U/Th systematics. As the age range considered here is very much shorter than the half-lives of ^238^U, ^235^U and ^232^Th, their decay can be considered as being linear with time. This greatly simplifies calculations of the abundances over time of the long-lived nuclides ^234^U and ^230^Th in the ^238^U decay chain and the yields of ^4^He.

(1) $$N^{238}U_{t}=N^{238}U_{init}e^{-\lambda_{238}t}\approx \;N^{238}U_{init}\;\left(1-\;\lambda_{238}t\right)$$

(Amounts *N* of isotopes are expressed in micromoles, µmol). Thus, the amount of ^4^He produced by the α-decay of ^238^U to ^234^Th followed by the double β-decay to ^234^U is given by Eq. 2:

(2) $$N{}^{4}He_{238U,t}\approx N{}^{238}U\times \lambda_{238}t$$

If the initial activity ratio (^234^*U*/^238^*U*)*_init_* ≠ 1, its value approaches equilibrium over time:

(3) $${\left({}^{234}U/{}^{238}U\right)}_{t}=\;\left[{\left({}^{234}U/{}^{238}U\right)}_{init}-1\right]\;e^{-\lambda_{234}t}+1$$

And (^234^*U*/^238^*U*)*_init_* is found from the inverse:

(4) $${\left({}^{234}U/{}^{238}U\right)}_{init}=\;\left[{\left({}^{234}U/{}^{238}U\right)}_{m}-1\right]\;e^{\lambda_{234}t}+1$$

Where subscript *m* refers to the measured value. As *d*^4^*He_234U_*/*dt* = - *d*^234^*U*/*dt*, the amount of ^4^He produced by the decay of ^234^U is found by integrating the decrease of disequilibrium over time interval *t* expressed in (3), adding it to the amount that would be produced in equilibrium, and converting to µmol by multiplying by *N*^4^*He*_238_*_U,t_* and dividing the last term by λ_234_*t*, bearing in mind relation (2).

(5) $$N{}^{4}He_{234U,t}=N{}^{4}He_{238U,t}\left\{1+\left[{\left({}^{234}U/{}^{238}U\right)}_{init}-1\right]\frac{1-e^{-\lambda_{234}t}}{\lambda_{234}t}\right\}$$

The amount of ^4^He produced by the decay of ^230^Th (ignoring ^226^Ra) has two components. First, ^4^He produced by ^230^Th, as this is yielded over time by the decay of ^234^U, is

(6) $$N_{1}{}^{4}He_{230Th,t}=6N{}^{4}He_{238U,t}\left[{\left({}^{234}U/{}^{238}U\right)}_{init}-1\right]\frac{\lambda_{230}}{\left(\lambda_{230}-\lambda_{234}\right)t}\left[\frac{1-e^{-\lambda_{234}t}}{\lambda_{234}}-\frac{1-e^{-\lambda_{230}t}}{\lambda_{230}}\right]$$

Second, a correction for (^230^Th/^238^U)_init_ ≠ 1 (analogous to Eq. 5)

(7) $$N_{2}{}^{4}He_{230Th,t}=\;6N{}^{4}He_{238U,t}\left\{1+\left[{\left({}^{230}U/{}^{238}U\right)}_{init}-1\right]\frac{1-e^{-\lambda_{230}t}}{\lambda_{230}t}\right\}$$

(This is the only modification to the solution of [16] ). Then

(8) $$N{}^{4}He_{230Th,t}=N_{1}{}^{4}He_{230Th,t}+N_{2}{}^{4}He_{230Th,t}$$

Further, minor contributions from the decays of ^235^U and ^232^Th (also treated as linear, and ignoring the mostly short-lived intermediate nuclides in their chains) are:

(9) $$N{}^{4}He_{235U,t}=7N{}^{235}U\times \lambda_{235}t\;\text{and}\;N{}^{4}He_{232Th,t}=6N{}^{232}Th\times \lambda_{232}t$$

The total accumulated ^4^He from these decays is:

(10) $$N{}^{4}He_{total,t}=\;N{}^{4}He_{238U,t}+N{}^{4}He_{234U,t}+N{}^{4}He_{230Th,t}+\;N{}^{4}He_{235U,t}+\;N{}^{4}He_{232Th,t}$$

We have had the opportunity to compare outcomes of this solution with those obtained using an exact solution found by P. Vermeesch using a matrix exponential approach, kindly made available to us, in which ^238^U decay is not treated as linear. The fit is very close for a large range of initial (^234^*U*/^238^*U*) and (^230^*Th/*^238^*U*) values: At 1000 ka, this solution yielded just 0.12 ‰ more ^4^He than our method. This is due to ^238^U decay not being treated as linear in the exact solution.

In the case of Methods 1, 2 and 3, solving the equations requires initial (^234^*U*/^238^*U*) and (^230^*Th*/^238^*U*) values to be assumed or calculated (see Table 2).

In Method 3, (^230^*Th*/^238^*U*)*_init_* is calculated by first calculating the present day value (^230^*Th*/^234^*U*)*_t_* with the assumption (^230^*Th*/^238^*U*)*_init_* = 0, via the solution used in calculating U/Th disequilibrium ages:

(11) $${\left({}^{230}Th{/}^{234}U\right)}_{t}=\frac{1-e^{-\lambda_{230}t}}{{\left({}^{234}U{/}^{238}U\right)}_{m}}+\left(1-\frac{1}{{\left({}^{234}U{/}^{238}U\right)}_{m}}\right)\frac{\lambda_{230}}{\lambda_{230}-\lambda_{234}}\left(1-e^{-\left(\lambda_{230}-\lambda_{234}\right)t}\right)$$

Then comparing the corresponding (^230^*Th*/^238^*U*)*_t_* value with the measured value (^230^*Th*/^238^*U*)*_m_* yields an excess value (^230^*Th*/^238^*U*)*_xs_*:

(12) $${\left({}^{230}Th{/}^{238}U\right)}_{xs}={\left({}^{230}Th{/}^{238}U\right)}_{m}-{\left({}^{230}Th{/}^{234}U\right)}_{t}\times {\left({}^{234}U{/}^{238}U\right)}_{m}$$

and

(13) $${\left({}^{230}Th{/}^{238}U\right)}_{init}={\left({}^{230}Th/{}^{238}U\right)}_{xs}\times e^{\lambda_{230}t}$$

In all these three methods, solution for age *t* is done by a commonly used successive approximation. A maximum and a minimum age (*t_max_* and *t_min_*) are set. Usually *t_min_* = 0 and *t_max_* is derived from the age obtained assuming full secular equilibrium:

(14) $$t_{max}=1.5\times \frac{N^{4}He_{m}}{8N{}^{238}U_{m}\;\lambda_{238}+7N{{}^{235}}_{m}\;\lambda_{235}+6N{}^{232}Th_{m}\;\lambda_{232}}$$

Where the factor 1.5 is introduced to ensure that *t_max_* is larger than the age obtained by any of the methods can possibly be. A starting value *t* is set as the average, *t* = (*t_max_ + t_min_*)/2, if the amount of ^4^He calculated is greater than the measured amount, then *t_max_* is set to the value of *t* used (while *t_min_* is not changed), and otherwise *t_min_* is set to it; then a new value for *t* is again set as the average and the calculation is repeated. In our work, a solution is achieved when the difference between the amounts of ^4^He calculated and measured is less than 10^−10^ µmol.

For Method 4, as in all U/Th age calculations, successive approximation of *t* by Eq. 11 is used, where the criterion for a solution is that the calculated value of (^230^*Th*/^234^*U*)*_t_* differs less than 0.0001 from the measured value (^230^*Th*/^238^*U*)*_m_* / (^234^*U*/^238^*U*)*_m_*. Since the use of this method is informed by *N*^4^*He* (indicating the presence of an old component, expected to be in or close to secular equilibrium, amounts of ^238^U, ^234^U and ^230^Th in equilibrium are first subtracted from the total U/Th budget using a correction factor *C_U_*, which is the fraction of ^238^U that resides in the old (detrital) component::

(15) $$C_{U}=\frac{{\left(N{}^{232}Th/N{}^{238}U\right)}_{m}}{{\left(N{}^{232}Th/N{}^{238}U\right)}_{D}}$$

Where subscript *D* refers to the old component. The denominator value can be set as the upper crustal average (=4) or a regional value from experience, or it can be derived by regression of (^230^*Th*/^238^*U*) vs. (^232^*Th*/^238^*U*) activity ratios if several samples are analyzed. The slope of such a regression yields the (^230^*Th*/^232^*Th*) ratio of the old component, which (assuming secular equilibrium of this component) allows to calculate its U/Th ratio. The corrected activity ratios (^234^*U*/^238^*U*)*_c_* and (^230^*Th*/^234^*U*)*_c_* to be substituted into Eq. 11 are then:

(16) $$\begin{array}{cc} {\left({}^{234}U/{}^{238}U\right)}_{c} & =\frac{{\left({}^{234}U/{}^{238}U\right)}_{m}-C_{U}}{1-C_{U}},{\left({}^{230}Th/{}^{238}U\right)}_{c}=\frac{{\left({}^{230}Th/{}^{238}U\right)}_{m}-C_{U}}{1-C_{U}}\;\text{and}\\ {\left({}^{230}Th/{}^{234}U\right)}_{c} & =\frac{{\left({}^{230}Th/{}^{238}U\right)}_{c}}{{\left({}^{234}U/{}^{238}U\right)}_{c}} \end{array}$$

An alternative way of determining (U,Th)-He ages using Methods 1 to 3 is by numerical integration of small time steps.

In Methods 1 and 2, for a time interval Δ*t* and with time going forward, the decays of the long lived isotopes are:

(17) $$\begin{array}{cc} \Delta^{238}U & =-N^{238}U\left(t\right)\times \lambda_{238}\Delta t,\Delta^{235}U=-N^{235}U\left(t\right)\times \lambda_{235}\Delta t\;\text{and}\\ \Delta^{232}Th & =-N^{232}Th\left(t\right)\times \lambda_{232}\Delta t \end{array}$$

And the changes in abundances for ^234^U and ^230^Th are:

(18) $$\begin{array}{cc} \Delta^{234}U & =N^{238}U\left(t\right)\times \lambda_{238}\Delta t-N^{234}U\left(t\right)\times \lambda_{234}\Delta t\;\text{and}\\ \Delta^{230}Th & =N^{234}U\left(t\right)\times \lambda_{234}\Delta t-N^{230}Th\left(t\right)\times \lambda_{230}\Delta t \end{array}$$

These changes augment and decrease *N*^234^*U*(*t*) and *N*^230^*Th*(*t*) over time, with the initial abundances of ^234^U and ^230^Th calculated from initial activity ratios input or (for (^234^*U*/^238^*U*) in Method 1) calculated via Eq. 4. In each time step, an amount Δ^4^*He* of ^4^He is produced:

(19) $$\Delta^{4}He=-\Delta^{238}U-\Delta^{234}U-6\Delta^{230}Th-7\Delta^{235}U-6\Delta^{232}Th$$

Which is a positive value. Over time, the abundances *N*^234^*U*(*t*) and *N*^230^*Th*(*t*) increase and/or decrease, while *N*^4^*He*(*t*) accumulates. When the point is reached where *N*^4^*He*(*t*) exceeds the measured amount of ^4^He, the stepping is stopped, and the age solution is taken as *t* - Δ*t*/2. For Method 2 this calculation therefore does not require successive approximation, while in Method 1, the initial (^234^*U*/^238^*U*) value is age dependent via Eq. 4 and approximation is done as for the analytical solutions. Convergence is achieved when the difference between successively obtained ages is equal or smaller than the age step.

For Method 3, the stepwise age calculation is done with time running backwards: Eqs. 17 and 18 are applied with inverted signs, so that Δ^4^*He* as calculated from Eq. 19 becomes a negative value: 4He is being consumed. Starting from the measured abundances of ^234^U, ^230^Th and ^4^He, the stepwise calculation is run until the point where *N*^4^*He*(*t*) is negative. As above, the age solution is then *t* - Δ*t*/2 and from the abundances *N*^234^*U*(*t*) and *N*^230^*Th*(*t*) at this last step, the initial (^234^*U*/^238^*U*) and (^230^*Th*/^238^*U*) activity ratios are obtained:

(20) $$\begin{array}{cc} {\left({}^{234}U{/}^{238}U\right)}_{init} & =\left[N^{234}U\left(t\right)/N^{238}U\left(t\right)\right]\times \left(\lambda_{234}/\lambda_{238}\right)\;\text{and}\\ {\left({}^{230}Th{/}^{238}U\right)}_{init} & =\left[N^{230}Th\left(t\right)/N^{238}U\left(t\right)\right]\times \left(\lambda_{230}/\lambda_{238}\right) \end{array}$$

The difference in ages obtained by the stepwise and analytical approaches is very small. With a time-step value Δ*t* = 2ka, it can reach about 3% if the initial disequilibrium (especially for ^230^*Th*/^238^*U*) is large but becomes < 1% at about 150 ka. Obviously as time steps are made smaller, the stepwise results come closer to the analytical ones.

In view of the unexpected results for initial (^230^*Th*/^238^*U*) of [1] in particular, it has been important to do the calculations using these completely different approaches to validate the solutions.

Since direct error propagation for these procedures is difficult, we use a standard Monte Carlo method of 500 calculations. For each repeated calculation *i*, a value is assigned to each measured parameter *P* as well as the decay constants that conforms to the Gaussian probability distribution. To do this, a random number *R* between 0 and 1 is generated and then a normal-inversion is applied to this, yielding a variable *Y* (which is negative if *R* < 0.5 and positive if *R* > 0.5). Then *Y* is scaled to the standard error σ*_P_* of the parameter, yielding a value $P_{i}^{*}$ which is then used in calculation *i*:

(21) $$P_{i}^{*}=P+Y\sigma_{P}$$

Then the mean and standard deviation of the 500 results are calculated; reported uncertainties are 2SD, i.e. 95% confidence. A result obtained from the mean values of the parameters is compared with the mean of the Monte Carlo results, to assess whether the uncertainties are symmetrical. For (U,Th)-He results, they always are, while U/Th ages (Method 4) give asymmetrical uncertainties when ages are older than about 300 ka.

Calculations are done using two alternative programs written in Microsoft Visual Basic 6 SP4 (one for the stepwise, and one for the analytical approach).
